# Negative relationship between brain-derived neurotrophic factor (BDNF) and attention: A possible elevation in BDNF level among high-altitude migrants

**DOI:** 10.3389/fneur.2023.1144959

**Published:** 2023-04-11

**Authors:** Jing Fan, Dongmei Chen, Niannian Wang, Rui Su, Hao Li, Hailin Ma, Fei Gao

**Affiliations:** ^1^Plateau Brain Science Research Center, Tibet University, Lhasa, China; ^2^Office of Safety and Health, Lhasa No. 1 Middle School, Lhasa, China; ^3^Beijing Key Laboratory of Behavior and Mental Health, School of Psychological and Cognitive Sciences, Peking University, Beijing, China; ^4^Academy of Plateau Science and Sustainability, People's Government of Qinghai Province, Xining, China

**Keywords:** brain-derived neurotrophic factor, attention, executive control, high altitude, Attention Network Test, event-related potential

## Abstract

**Objective:**

Brain-derived neurotrophic factor (BDNF), a member of the neurotrophic family that plays a vital role in regulating neuronal activity and synaptic plasticity in the brain, affects attention. However, studies investigating the association between BDNF and attention in long-term high-altitude (HA) migrants are limited in the literature. As HA affects both BDNF and attention, the relationship between these factors becomes more complex. Therefore, this study aimed to evaluate the relationship between peripheral blood concentrations of BDNF and the three attentional networks in both behavioral and electrical aspects of the brain in long-term HA migrants.

**Materials and methods:**

Ninety-eight Han adults (mean age: 34.74 ± 3.48 years, 51 females and 47 males, all have lived at Lhasa for 11.30 ± 3.82 years) were recruited in this study. For all participants, the serum BDNF levels were assessed using enzyme-linked immunosorbent assay; event-related potentials (N1, P1, and P3) were recorded during the Attentional Networks Test, which was used as the measure of three attentional networks.

**Results:**

Executive control scores were negatively correlated with P3 amplitude (*r* = −0.20, *p* = 0.044), and serum BDNF levels were positively correlated with executive control scores (*r* = 0.24, *p* = 0.019) and negatively correlated with P3 amplitude (*r* = −0.22, *p* = 0.027). Through grouping of BDNF levels and three attentional networks, executive control was found to be significantly higher in the high BDNF group than in the low BDNF group (*p* = 0.010). Different BDNF levels were associated with both orienting scores (χ^2^ = 6.99, *p* = 0.030) and executive control scores (χ^2^ = 9.03, *p* = 0.011). The higher the BDNF level, the worse was the executive function and the lower was the average P3 amplitude and vice versa. Females were found to have higher alerting scores than males (*p* = 0.023).

**Conclusion:**

This study presented the relationship between BDNF and attention under HA. The higher the BDNF level, the worse was the executive control, suggesting that after long-term exposure to HA, hypoxia injury of the brain may occur in individuals with relatively higher BDNF levels, and this higher BDNF level may be the result of self-rehabilitation tackling the adverse effects brought by the HA environment.

## 1. Introduction

Brain-derived neurotrophic factor (BDNF), a member of the family of secreted proteins that support the growth, survival, and differentiation of neurons (1), is vital for regulating synapses and synergistic interactions between neuronal activity and synaptic plasticity in the brain (2). BDNF can enhance neurorestorative effects in the prefrontal cortex and hippocampus, including newborn cell proliferation and survival, granule cell neurogenesis, synaptogenesis, and increased dendritic integrity (3, 4). Because cognitive functions such as learning, memory, and attention rely on the operation of the prefrontal cortex and hippocampus (5, 6), when the BDNF level is reduced or when BDNF activity is compromised in the prefrontal cortex and hippocampus, dysfunction of these cognitive functions might occur. Previous studies have reported that BDNF level in peripheral blood is associated with cognitive functions, that peripheral BDNF levels were positively correlated with performance in tasks measuring working memory and negatively correlated with the severity of learning impairment (7, 8), and that external supplementation of BDNF can reverse learning deficits in hippocampal neurons (9). More studies have found that BNDF is associated with attention and that decreased BDNF activity can cause dysfunction in midbrain dopaminergic, neuronal migration, and neuronal plasticity (10, 11). This further leads to attention-deficit hyperactivity disorder (ADHD) (12), a mental disorder characterized by generalized and developmentally unbefitting inattention (11, 12). Moreover, other studies have found that an increase in BDNF level may be the reason for the improvement in attentional function (13, 14). However, other researchers found no correlations between serum and plasma levels of BDNF and attention in any measure (15).

Based on the above evidence, inconsistencies have been noted in the association between BDNF and attention. Attention is a fundamental function of cognition, which refers to a system that selectively concentrates on a discrete aspect of information while ignoring other perceivable information (16). Fan proposed dividing the attentional process into the following three diverse but interrelated networks: alerting, orienting, and executive control. To better study each attention process, the Attentional Network Test (ANT), a particular test combining the cue-target paradigm and Flanker task, has been designed to measure these three subdivisions of attention (17), and different attention systems were found to have different neural bases that are related to BDNF. Alerting the sense that produces and maintains optimal vigilance and performance in the course of tasks is the early stage of attention (18). The activation region of alerting is mainly distributed in the dorsolateral prefrontal cortex (19), which can be disturbed without persistent network activity mediated by the synergy of BDNF and its receptor tropomyosin-related kinase B (TrkB) (20). Orienting is a network that emphasizes on the ability to prioritize sensory input by selecting a modality or location (18), and the main activation regions of orienting are in the parietal and frontal cortex and the temporoparietal junction area of the right hemisphere (21). Executive control is the dominant network that represents the ability to accomplish target tasks by inhibiting interference and resolving conflicts among thoughts, feelings, and behaviors (22). According to previous brain imaging studies, the executive control network was mainly distributed in the anterior cingulate gyrus and dorsolateral prefrontal cortex (23), and BDNF can sustain neuronal survival in these regions by enhancing the cannabinoid receptor 1 (CB1R) transcripts (24, 25). The above evidence suggests that attention is a series of processes and that BDNF is related to the brain areas concerning these attentional networks. Therefore, this study speculates that the association between BDNF and attention in previous studies is actually the association between BDNF and parts of the whole attention process, as the different tasks used in these studies might only access one or some parts of the whole attention process. Zhang et al. explored the relationship between BDNF and attention networks using the ANT paradigm and found that BDNF was differentially associated with attention sub-networks, with lower BDNF levels being suggestive of poorer orientation and executive functioning, whereas no such results were found for alerting (26).

The human brain is highly sensitive to oxygen supply and can be easily affected at high altitude (HA) (27–29), an environment characterized by hypoxia (30). So that the dependence of attention function on the brain makes it susceptible to HA. Among the 4,200-m residents, the orienting function declined, but the executive control improved, while in the 2,438.4-m simulated facility, superior efficiency was noted in orienting compared with that noted at sea level (26, 31). In another study, alerting was significantly declined in the HA cohort than in the low-altitude (LA) cohort, but no significant differences were noted in orienting or executive control between the HA and LA groups (32). Event-related potential (ERP), which originates from electroencephalography, can offer temporal information about attentional processes (33). Moreover, evidence has been found in ERP studies, further testifying that HA can influence attentional functions. The N1 and P1 components, indicators of alerting and orienting, were found to be declined at HA (34, 35). P3, the marker of executive control (36), was found to be declined in one study and improved in another study in an HA environment (26, 35). These facts indicate inconsistency in results reported for attention variations when exposed to HA.

The above evidence suggests that in the HA environment, different experimental settings, such as exposure duration and altitude, cause different changes in brain nerves, resulting in different changes in attention function. BDNF, a prominent factor associated with attention function, may also play a role in the inconsistency of attention variation in HA. BDNF level can be reportedly elevated by both acute hypoxia and intermittent hypobaric hypoxia to promote neurogenesis through BDNF-mediated signaling (1, 37, 38), while chronic hypoxia can result in a decrease in BDNF level (39). An increase in BDNF can improve attention impairment and some studies have found that increased BDNF levels are associated with inattentive problems (40, 41). It is worth noting that attention dysfunction may represent the presence of brain damage (42), which can induce an increase in BDNF to promote neuronal survival and reverse brain damage (43–45). In HA, both BDNF and attention can be affected, and there is an interaction between them. This bi-directional influence makes the association between them at HA more complicated than that at sea level; therefore, inconsistency in variations of attention function have been noted. However, only few studies have been conducted to explore the relationship between BDNF and attention function in the HA environment, especially on the relationship between the three attention sub-networks measured by the ANT paradigm and BDNF. To further understand the relationship between attention and BDNF in the HA environment, this study used the ERP technique, time-frequency representation (TFR) analysis technique, and ANT paradigm to analyze the relationship between BDNF expression and attentional function in both behavioral and EEG aspects of long-term HA migrants through a cluster analysis method to classify the attentional function performance of migrants exposed to HA environments for a long time and evaluate the difference in attentional networks in HA by BDNF level.

## 2. Materials and methods

### 2.1. Participants

**Figure 1:**
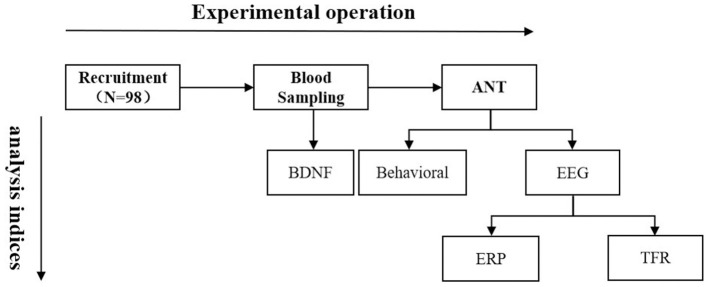
Flow chart of the experiment. ANT, Attention Network Test; BDNF, brain derived neurotrophic factor; EEG, electroencephalo-graph; ERP, event-related potential; TFR, time-frequency representation.

A total of 98 Han adults in Tibet were recruited (51 females, mean age 35 ± 3.65 years; 47 males, mean age 34.74 ± 3.48 years). All of them have lived in Lhasa for 11.30 ± 3.82 years (4.57–21.08 years), with 10.12 ± 0.66 months each year (9–12 months). Each of them was born in an LA area and have never been to any HA places other than Lhasa before they moved to Lhasa to work and live, and the first arrival to Lhasa occurred after adulthood. The participants had normal or corrected vision, no color impairment, no history of mental illness, traumatic brain injury, hypertension, heart disease, or other major diseases. This study adhered to the guidelines of the Declaration of Helsinki and was approved by the Government of the Tibet Autonomous Region and the Local Ethics Committee of Tibet University. All participants joined the experiment voluntarily, signed informed consent forms before the experiment, and received certain remuneration after the experiment. The whole process of the experiment is displayed in Figure 1.

### 2.2. Measurement of BDNF

Fasting venous blood samples from 98 participants were collected at the Faking Hospital of Lhasa, and the collectors and analysts were qualified or professionally trained. The venous blood samples were coagulated for 30 min and then separated at 15°C by high-speed centrifugation for 15 min to obtain the serum, which was stored at −20°C until analysis. BDNF level was measured using enzyme-linked immunosorbent assay with The Quantikine^®^ Total BDNF Immunoassay (Catalog: DBNT00, SBNT00, PDBNT00, R&D Systems, Minneapolis, Minnesota, USA), and the parameters for analysis were: absorbance as 450 nm, calibration wavelength as 570 nm.

### 2.3. Collecting and analysis of data from Attention Network Test

**Figure 2:**
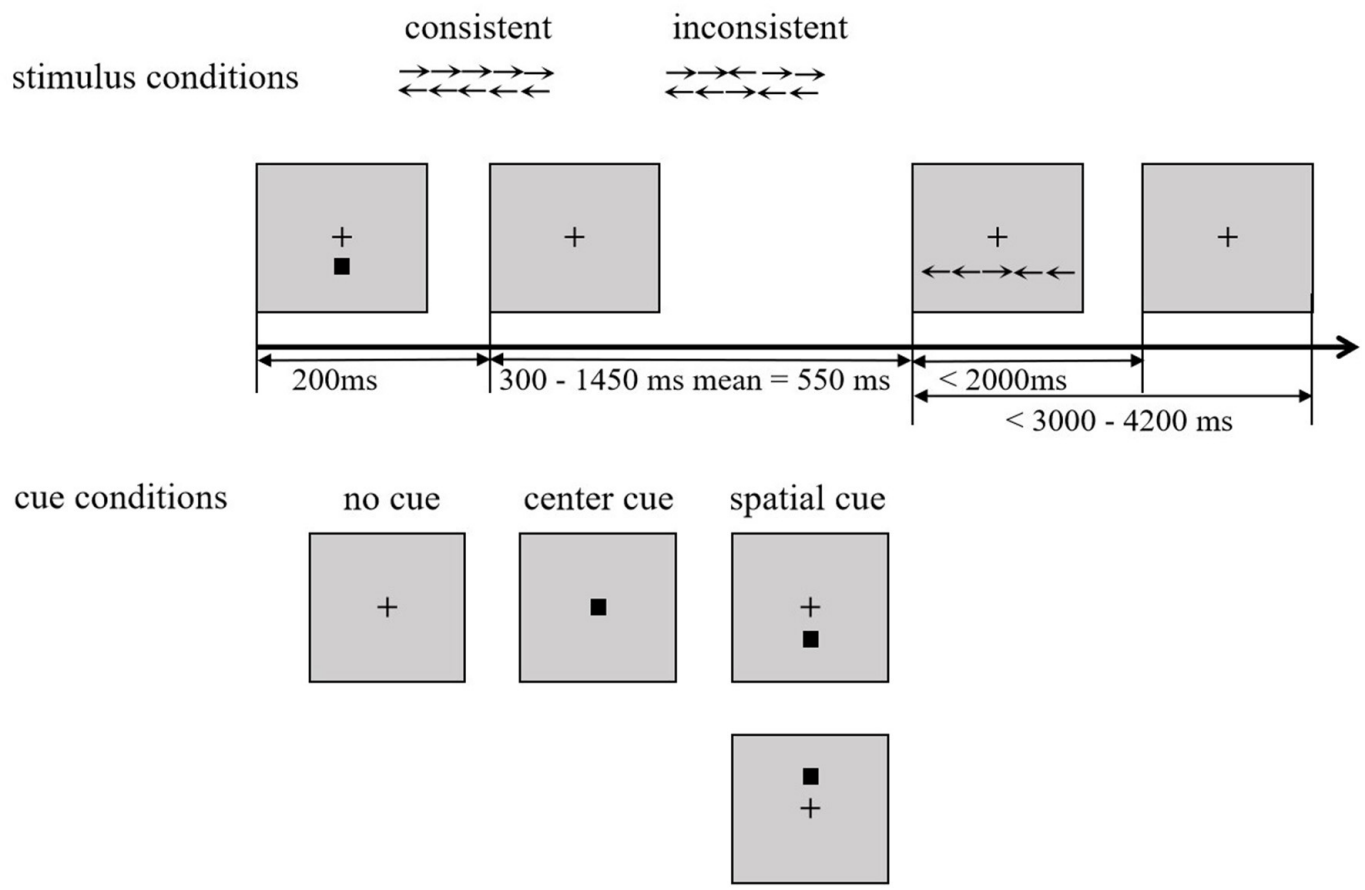
Flow chart of ANT. ANT, Attention Network Test.

The Attention Network Test (ANT) paradigm was adopted in this study to measure the efficiency of the three brain attention network functions (alerting, orienting, and executive control). The ANT paradigm was programmed using E-Prime 2.0 (Carnegie Mellon University, University of Pittsburgh and Psychology Software Tools, Inc, Pittsburgh, USA) and analyzed using IBM^®^ SPSS^®^ Statistics 27 software (SPSS, International Business Machines Corporation, New York, USA). The details are shown in Figure 2, wherein the paradigm included three cue conditions (no cue, central cue, and spatial cue) and two stimulus conditions (consistent and inconsistent). In the consistent stimulus condition, five arrows were pointing in the same direction, whereas in the inconsistent stimulus condition, the arrows (3rd) in the center faced opposite directions with the arrows on both sides (1st, 2nd, 4th, and 5th). If there are clues, they will appear before the target stimulus is presented. In the center cue condition, the cue covered at the fixation point with only alerting included, while in the spatial cue condition, the cue appeared above or below the central fixation point with both alerting and orienting included. All the stimuli have a black background, the stimulus material is white, each small arrow has a viewing angle of 0.58°, and the distance between adjacent arrows is 0.06°. The whole target is presented as five arrows with a viewing angle of 3.27°, and the arrows have two orientations, 1.06° from the central fixation point and below or above the central fixation point. All trials were designed in a modular manner, and the probability of occurrence of the six conditions in each module was the same, including two stimulus types (consistent and inconsistent) and three cues (no cue, central cue, and spatial cue). There were six modules in the experiment, and each module comprised 108 trials. Stimulus conditions and cue conditions were combined to presented in each trail and the frequency of each stimulus conditions and cue conditions occurred equally. The participants were instructed to sit quietly in front of the experiment computer and to determine the direction of the central arrow, with the “F” key corresponding to the middle arrow pointing left and the “J” key to the middle arrow pointing right in each trail. The practice modules were presented before the formal experiment.

The ANT Neuro device was used to record the electroencephalographic (EEG) and DC samples according to the extended 64 conducting caps of the International 10–20 system. The signal was recorded at a sampling rate of 500 Hz, using CPz as an online reference. The scalp resistance at the electrode was kept below 5 Kω, the filter band pass was kept at 0.05–100 Hz, and the sampling rate was kept at 500 Hz.

The reaction time (RT) of the six conditions was measured using the ANT paradigm, and data other than ±3 SD were excluded (excluded number: 0). The effect values of the three sub-networks were calculated according to the calculation formula of attention network efficiency as follows:

Alertness effect = RT_nocue_ – RT_centralcue_, wherein the higher the value, the better promotion effect of the center cue, the higher the alertness efficiency; Orientation effect = RT_centralcue_ – RT_spatialcue_, wherein the larger the value, the higher the orientation efficiency; Executive control effect = RT_inconsistentstimulus_ – RT_consistentstimulus_, wherein the smaller the value, the higher the executive control efficiency.

### 2.4. Collection and analysis of EEG data

Matlab R2016b and EEGLAB toolbox were used for data preprocessing, and the EEG signal was filtered at 0.1–40 Hz and converted to bilateral mastoid mean reference offline. The duration of the ERP analysis was 200 ms before and 1,000 ms after the presentation of the target stimulus, and the baseline correction was 200 ms before the presentation of the stimulus. Independent Component Analysis was used to remove the horizontal and vertical electroencephalograms and artifacts, and other artifacts were removed manually. Finally, spurious signals with amplitudes of ±100 μV were excluded.

**Figure 3:**
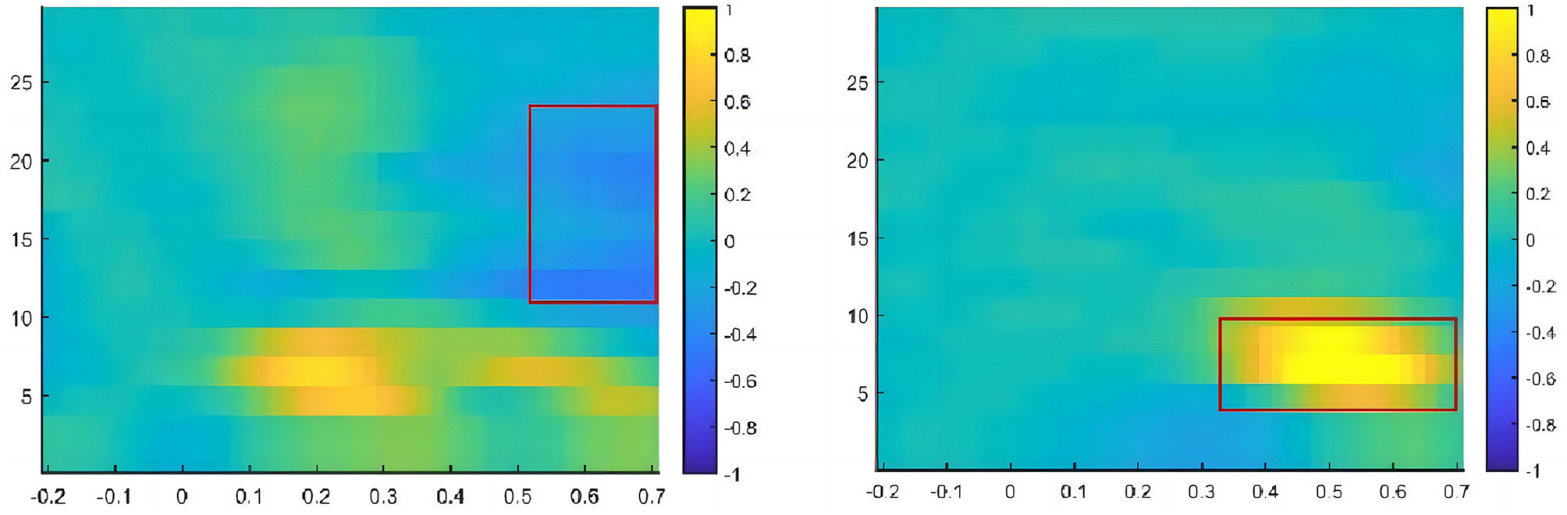
Schematic diagram of EEG components. EEG components, electroencephalographic components.

The ERPLAB toolbox was used for component processing. The conditions before and 100 ms after the presentation of the target stimulus and the selected time window are shown in Figure 3. The time window for the calculation of the P1 component was 100–150 ms. The superimposed average values of this component on the parietal lobe (P3, Pz, and P4) and the occipital lobe (O1, Oz, and O2) electrodes were used to analyze alertness and orientation. The time window of the N1 component calculation was 150–230 ms, and the superimposed average of the component was calculated for the parietal lobe (P3, Pz, and P4) and the occipital lobe (O1, Oz, and O2) electrodes to analyze alertness and orientation. The P3 component was analyzed under consistent and inconsistent conditions, and the calculated time window was 620–720 ms. Stacking averages were calculated for the electrodes of the component in the middle line (Fz, Cz, FCz, Fp1, Fp2, and Fpz) to analyze the executive control function.

### 2.5. Analysis of time-frequency data

**Figure 4:**
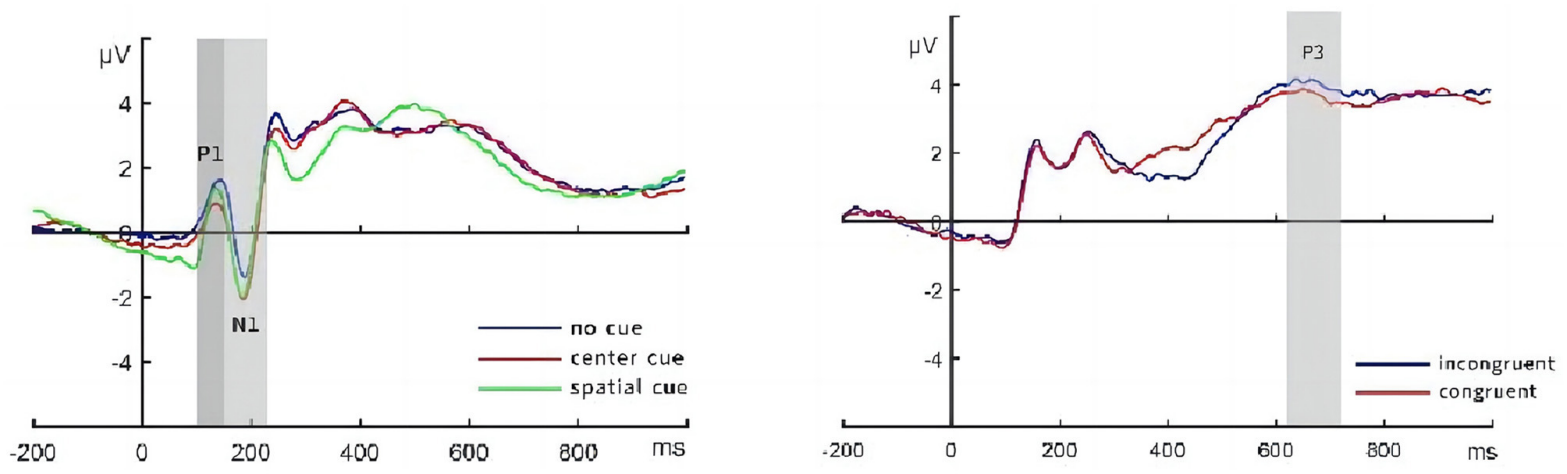
Time window and frequency band for time-frequency analysis.

MATLAB R2017a and the FieldTrip toolbox were used for the time-frequency analysis. The short-time Fourier Hanning window analysis method was used in this study, with a time window of 200–700 ms, frequency range of 1–30 Hz, and step size of 1 Hz. Based on the total mean map and differential wave topographic map, the TFR results of each electrode, and relevant literature, we defined the following three regions of interest in the directed network: parietal lobe (P3, Pz, and P4), occipitotemporal lobe (PO3, PO4, PO7, and PO8), and occipital lobe (O1, Oz, and O2). The beta band (12–25 Hz) in the 500–700-ms time window was analyzed. The electrodes of the executive control network were set as Fz, FCz, Cz, and Pz, and the theta frequency band (4–7 Hz) in the 300–700-ms time window was analyzed (Figure 4).

### 2.6. Data analysis

The statistical analyses were conducted using IBM^®^ SPSS^®^ Statistics 27 software. Pearson's correlation Percentile grouping was used to group different levels of BDNF and attention functions scores, two-factor analysis of variance (ANOVA) was conducted with gender (male, female) and BDNF groups (low and high) as between-subjects factors to test the effect of gender and different BDNF levels on behavior and EEG. Pearson's chi-square tests were used to further determine the associations between attention functions and BDNF.

## 3. Results

### 3.1. Relationship between behavior, EEG, and BDNF

**Figure 5:**
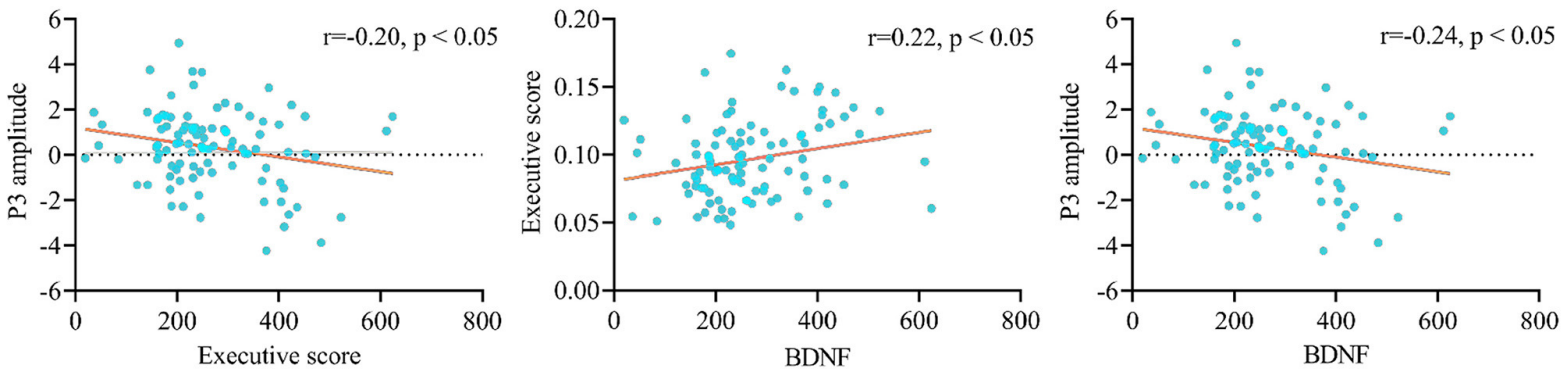
Correlations between executive control, P3 amplitude and BNDF. BNDF, brain-derived neurotrophic factor.

In the correlation analysis of attention function and EEG components and frequency band energy, only the executive control score and P3 wave were negatively correlated (*r* = −0.20, *p* = 0.044); that is, the better the executive function, the greater the amplitude of the P3 wave. Other attentional networks were not correlated with EEG components or frequency band energy. Correlation analysis of attention function and BDNF showed that the executive control score was positively correlated with BDNF (*r* = 0.24, *p* = 0.019) (Figure 5), that is, the higher the BDNF level, the worse the executive function, while the correlations between alerting (*r* = −0.02, *p* = 0.850), orienting (*r* = −0.03, *p* = 0.800) and BDNF were not significant. Correlation analysis between the mean amplitude of P3 and BDNF showed that the mean amplitude of P3 was negatively correlated with BDNF (*r* = −0.22, *p* = 0.027) (Figure 5); that is, the higher the BDNF level, the lower the mean amplitude of P3. BDNF had no correlation with other EEG components or frequency band energy.

### 3.2. Effect of sex and BDNF on attention network/EEG component/time-frequency energy

BDNF levels were grouped according to the percentile grouping approach. Overall, 0%−30% of the participants were in the low BDNF group, 30%−70% were in the medium BDNF group, and 70%−100% were in the high BDNF group (46, 47). Table 1 shows the basic information of participants in the three BDNF groups and only low and high groups (*N* = 58) were used in the following ANOVA and Pearson's chi-square test analysis (48, 49).

**Table 1:** Basic information of participants in the high and low BDNF groups.

**BDNF group**	**BDNF levels (pg/ml)**	**Male (*n*)**	**Female (*n*)**	**Age (years)**	**Time in Tibet (years)**
Low	150.58	11	18	35.08 ± 3.42	11.72 ± 3.48
Medium	248.52	20	20	34.87 ± 3.90	11.17 ± 4.11
High	412.63	16	13	34.67 ± 3.26	11.06 ± 3.84

BNDF, brain-derived neurotrophic factor.

**Figure 6:**
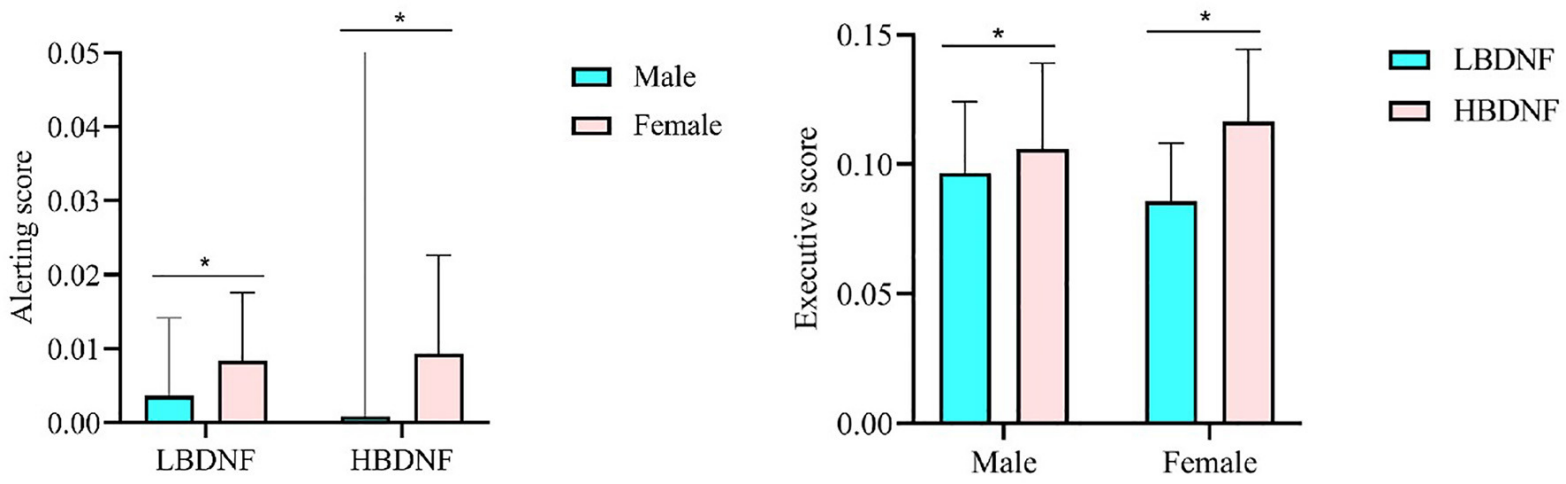
The gender effect in alerting and the BDNF effect in executive control. *p < 0.05. BNDF, brain-derived neurotrophic factor.

ANOVA was used to analyze the influence of sex and high/low BDNF group (hereinafter, referred to as “group”) on the indicators of attention network/EEG component/time-frequency energy. The results are presented in Table 2 and Figure 6. The results showed that the main effect of gender on alertness function was significant and that the alertness scores of males were significantly lower than those of females (*p* = 0.023). Executive control scores in the high BDNF group were significantly higher than those in the low BDNF group (*p* = 0.010). In other attentional networks, EEG components and time-frequency main effects were not significant, and the interaction was not significant.

**Table 2:** Two-factor ANOVA of network/EEG component/time-frequency energy.

	**Gender**			**BDNF group**			**Gender** ^*^ **BDNF group**		
	*F*_(1, 54)_	***p***	***MS***	*F*_(1, 54)_	***p***	***MS***	*F*_(1, 54)_	***p***	***MS***
Alerting^*^	5.50	0.023	0.001	0.10	0.758	<0.001	0.45	0.505	<0.001
Orienting	0.56	0.457	<0.001	0.11	0.738	<0.001	0.68	0.412	<0.001
Executive control^*^	0.00	0.972	<0.001	7.15	0.010	0.01	2.01	0.162	<0.001
N1 orienting	2.52	0.118	3.68	0.003	0.885	0.05	0.08	0.781	0.11
P1 alert	0.34	0.563	0.31	1.38	0.245	1.25	0.16	0.696	0.14
P1 orienting	2.05	0.158	3.48	0.30	0.585	0.51	0.09	0.762	0.16
N1 alert	1.08	0.303	0.81	3.01	0.089	2.25	0.11	0.747	0.08
P3 executive control	0.17	0.679	0.51	3.58	0.064	10.49	0.13	0.724	0.37
Theta	0.46	0.500	0.18	0.23	0.635	0.09	2.65	0.109	1.03
Beta	0.14	0.714	0.04	0.64	0.427	0.18	0.03	0.860	0.01

BNDF, brain-derived neurotrophic factor; EEG components, electroencephalographic components. ^*^Indicates the existence of a significant effect, *p* < 0.05.

Based on the results above, to further determine the relationship between executive function and BDNF expression, executive control scores were grouped like BDNF grouping. Pearson's chi-squared test was used to analyze the correlation between BDNF levels and executive control scores, and the results (Table 3) indicated that different BDNF levels were associated with executive control scores (χ^2^ = 9.03, *p* = 0.011). We also determined the relationship between alerting, orienting and BDNF expression through Pearson's chi-squared test and found different BDNF levels were associated with orienting scores (χ^2^ = 6.99, *p* = 0.030) too.

**Table 3:** Chi-square test of BDNF and three attentional networks.

**BDNF group**	**Total**	**Alerting group (%)**			**χ*^2^***	***p***
		**Low**	**Moderate**	**High**		
**Chi-square test of BDNF and alerting**						
Low	29	8 (44.44)	13 (52.00)	8 (53.33)	χ^2^ = 0.33	*p =* 0.848
High	29	10 (55.56)	12 (48.00)	7 (46.67)		
**BDNF group**	**Total**	**Orienting group (%)**			χ*^2^*	***p***
		**Low**	**Moderate**	**High**		
**Chi-square test of BDNF and orienting**						
Low	29	8 (44.44)	18 (66.67)	3 (23.08)	χ^2^ = 6.99	*p =* 0.030
High	29	10 (55.56)	9 (33.33)	10 (76.92)		
**BDNF group**	**Total**	**Executive control group (%)**			χ*^2^*	***p***
		**Low**	**Moderate**	**High**		
**Chi-square test of BDNF and executive control**						
Low	29	11 (64.71)	13 (65.00)	5 (23.81)	χ^2^ = 9.03	*p =* 0.011
High	29	6 (35.29)	7 (35.00)	16 (76.19)		

BNDF, brain-derived neurotrophic factor.

## 4. Discussion

In this study, ANT and ERP techniques were adopted to focus on the relationship between the level of BDNF and three attentional networks among individuals exposed to long-term HA environments. Through correlation analysis, executive control was found to be positively correlated with the P3 amplitude and the BDNF level was negatively correlated with executive control and the P3 amplitude. This means that executive control in the behavioral aspect is consistent with the electrical aspects of the brain, wherein the higher the BDNF level, the worse the executive function. In addition, executive control scores were grouped into three groups according to the percentile grouping approach and were found to be associated with different BDNF levels, indicating that individuals with worse executive function have relatively higher BDNF levels. These findings imply that the relationship between BDNF and attentional function is bi-directional. Further, using two-factor ANOVA, a gender difference in alerting was found, indicating that females had better alerting function than males after long-term HA exposure.

The main result of this study was the positive relationship between executive control and the P3 amplitude and the negative relationship between BDNF and both executive control and the P3 amplitude. This depicts that after long-term HA exposure, individuals with higher BDNF levels have worse executive function in both behavioral and ERP aspects. First, the relationship between executive control and the P3 amplitude demonstrated an association between attentional behavior and brain activity, as reported previously (50). Attention, especially executive function, has long been proven to be a brain function that relies on the activation of the frontal lobe (51, 52). The ERP components N1, P1, and P3, measured by the average of the electrodes of the component in the parietal and occipital lobes and the middle line, which mostly represent the frontal lobe (35), have been found to be indicators of attentional sub-networks. N1 indicates the function of alerting, P1 indicates the function of orienting, and P3 is an indicator of executive control (34–36). These facts prove the strong association between attention function and the frontal brain. Recently, an increasing number of studies have used ERP components to represent attention function to examine its association with other factors (26, 35, 53). Although no correlations of alerting and orienting have been found with N1 and P1 in this study, we should not rush to the conclusion that alerting and orienting in behavioral aspects do not have associations with corresponding brain regions for consideration of all the facts found in previous studies.

Based on the result that executive control and P3 were positively correlated, the present study also found a negative association between BDNF and both executive control and the P3 amplitude, indicating that the higher the BDNF level, the poorer the executive control. These results are inconsistent with the findings of most previous studies, which mainly reported a positive relationship between BDNF and cognition function (7, 9). BDNF is involved in neuronal growth and survival; it regulates synapses and synergistic interactions between neuronal activity and synaptic plasticity (1, 2) and is considered to be protective for attention function. Lower BDNF levels were reported to be correlated with executive control dysfunction, which was measured using verbal fluency tests and Wisconsin card sorting tests in patients with schizophrenia (54, 55). Based on these findings, the initial hypothesis of the present study was that BDNF expression at HA is positively correlated with attentional networks and corresponding ERPs components. However, the present study analyzing this association found the opposite outcome, wherein a negative association was found between executive control and BDNF, revealing that individuals with higher BDNF levels tend to have poorer executive control functions. Although the negative result was somewhat unexpected, the present study is not the first to draw such a conclusion, and the negative correlation has merits. In 1999, Croll et al. (56) found that over-expressed BDNF transgenic mice showed significant impairment in learning and hyperexcitability in the CA3 area of the hippocampus, suggesting that excessive BDNF expression interferes with memory and learning, similar to that reported in human children (15). These results demonstrate that BDNF levels may be negatively related to cognitive function. The negative association was explained by the fact that the higher expression of BDNF can cause overall membrane hyperexcitability in brain structures, and the hyperexcitability is toxic to normal plasticity (56) and further leads to abnormalities in brain function. However, this explanation is not suitable for the negative association between BDNF and attention with a long-term exposure of hypoxia, as it's been reported that elevation in BDNF level often occurs when the exposure time to HA is short (37). Long-term exposure to hypoxia, as previous studies have observed that BDNF levels were significantly decreased after an exposure of hypoxia of around 2.3 years compared to sea level (39). In this work, we describe that after 11 years of hypoxia mean BDNF levels are 268.1 ± 115.4 pg/ml, which is lower than after 2.3 years of hypoxia or at sea level. Therefore, the negative association of BDNF with attention is unlikely to be caused by abnormally increased BDNF levels.

As correlation does not imply a causal relationship, we not only found higher executive control scores (worse executive control function) in people with higher BDNF levels in this study but also noted that different BDNF levels were associated with different orienting scores and executive control scores. This indicates that, there could be a two-way interaction between BDNF and attention, besides the normal thinking that the BDNF can affect attention, attention function could also induce the change of BDNF level. For its characteristics on neurons and synapses, BDNF has been discovered to play a momentous role in neuronal survival and synaptogenesis that occur in reparative processes after traumatic brain injury (45). Approximately 1–6 h after traumatic brain injury, BDNF level reportedly increased maximally in the ipsilesional hippocampus (43), whereas Matzilevich et al. (57) found increased BDNF expression at 26 h after brain trauma, and the increase in BDNF mRNA expression in the hippocampus can be sustained for 2 weeks after injury, as reported previously (44). This evidence suggests that brain injury could elevate BDNF expression for self-rehabilitation by mimicking the endogenous protective response of the brain. It has been found in previous studies that hypoxia can cause injuries in several brain areas and dysfunctions in cognition performance can be an external manifestation of brain injury (58, 59), so we suspected poorer executive function could represent more injuries of the brain at HA in this study. Individuals with worse executive function may have more endogenous protective response in the brain by elevating BDNF level, making the negative association between BDNF and attention function reasonable. However, other researchers have also discovered different cases, wherein brain injury may not exert a significant influence on BDNF expression in some cases (60), while some researchers found decreased BNDF expression after injury (61, 62). Therefore, this mechanism is only one possible reason and is not a firm conclusion that BDNF and attention function are negatively associated.

Apart from the main result that worse executive function is associated with higher BDNF levels, this study found a gender difference in alerting: males have lower scores than females, indicating that females have better alerting function than males after long-term HA exposure. Alerting is the sense that produces and maintains optimal vigilance and performance in the course of tasks and the early stage of the attentional process (18). Jung et al. (63) conducted a meta-analysis of studies on the effects of hypoxia on cognition in HA and found gender differences in this effect; males showed significant cognitive impairment compared with females. A possible explanation could be that females have relatively higher peripheral oxygen saturation (SpO_2_) and estrogen hormone levels than males, and both of the factors have neuroprotective effects and provide greater resistance to hypoxia. However, as no study has investigated the hypoxia-cognition interaction in women during a specific timing of the menstrual cycles (estrogen levels are lower than usual), the protective effect of estrogen hormones should be considered with caution and needs to be further verified by discovering whether cognitive functions are declined in females who are in the menstrual cycles under hypoxia. Moreover, previous studies have found that hypoxia can cause oxidative stress and that males tend to be more susceptible to oxidative stress compared with females (64, 65). As oxidative factors can contribute to depression, individuals with depression are prone to have an attentional bias (66, 67). It can be suspected that hypoxia is partially involved in causing more impairment in attentional function among males than among females with oxidative factors, with depression being the intermediary factor.

### 4.1. Limitations and prospects

Limitations also appear in this study. This study does not have longitudinal data, and the lack of BDNF and attention data at sea level and other time periods of hypoxia exposure, makes it impossible to determine the time effect of the association between BDNF and attention function. Previous studies found the influence of long-term hypoxia exposure on cognitive function exists in two types of circumstances. Cognition may continue to be damaged or it could return to an undamaged state owing to the offset effects of the adaptive process (68), the lack of sea level data making it hard to draw firm conclusion of the variations of attention function. However, the results of the present study show that individuals with poorer executive function have higher BDNF levels. Revealing to us that individuals with relatively worse functions may be still go through adapting, while individuals with better functions have already completed the adaptive process. Therefore, to more comprehensively understand the relationship between BDNF and attention at different hypoxia exposure times, the mechanism of association between BDNF and executive control in short-term migrants and the mechanism of the negative correlation between BDNF and executive control in long-term migrants, future studies should consider the BDNF level in participants at sea level, the early days, and various periods of HA exposure if experimental conditions permit.

### 4.2. Conclusions

In summary, this study used ANT and ERP techniques to explore the relationship between BDNF and attentional functions (namely alerting, orienting, and executive control) among long-term HA migrants. We found that executive control is positively correlated with the P3 amplitude and that both these factors are negatively correlated with BDNF level, implying that the worse attention function, the higher the BDNF level in HA migrants, which may be a self-rehabilitation. In addition, females had better alerting function than males, as females may experience fewer negative effects of hypoxia. To the best of our knowledge, this study is the first to explore the relationship between BDNF and the three attentional networks among long-term HA migrants, providing a more comprehensive view of BDNF and attention function under long-term hypoxia.

## Data availability statement

The raw data supporting the conclusions of this article will be made available by the authors, without undue reservation.

## Ethics statement

The studies involving human participants were reviewed and approved by the Government of the Tibet Autonomous Region, the Local Ethics Committee of Tibet University. The patients/participants provided their written informed consent to participate in this study.

## Author contributions

HM and HL obtained funding, provided general guidance, and approved the final manuscript. RS and FG conceived and designed the experiment. NW and DC collected and analyzed the data. JF drafted the manuscript. All authors contributed to the manuscript and approved the submitted version.
